# Processing of veracity cues: how processing difficulty affects the memory of event description and judgment of confidence

**DOI:** 10.1186/s41235-025-00629-2

**Published:** 2025-05-28

**Authors:** Nicole Antes, Stephan Schwan, Markus Huff

**Affiliations:** 1 Leibniz-Institut Für Wissensmedien, Schleichstraße 6, 72076 Tübingen, Germany; 2 Department of Psychology, University of Tübingen, Schleichstraße 4, 72076 Tübingen, Germany

**Keywords:** Veracity cue, Truth bias, Confidence, Memory, Event description, Processing fluency

## Abstract

In today's rapid dissemination of information, discerning truth from falsehood is crucial. We investigated how cues signaling information veracity influence memory accuracy and confidence in coherent narratives. Two studies manipulated perceptual difficulty in distinguishing true-labeled from false-labeled information in event descriptions using font color as a cue. Study 1 (*N* = 198) revealed that the presence of veracity cues reduces memory accuracy for the event description. Study 2 (*N* = 248) showed that when differentiating veracity cues became more challenging, false-labeled information was more frequently misidentified and less accurately remembered. Confidence ratings decreased with the presence of veracity cues (Study 1) but resulted in disproportionately high confidence for sentences labeled as false that were confused as true (Study 2). False-labeled information was less retained, yielding initially more accurate event representation. However, once stored, false-labeled information was recalled with confidence as true, leading to a false representation. Therefore, mechanisms such as highlighting the veracity of information within coherent news articles on social media should be used with consideration.

**Supplementary Information:**

The online version contains supplementary material available at 10.1186/s41235-025-00629-2.

## Introduction

In today's constant flood of information, accelerated by social media, the representation of events often shapes perceptions of reality (Fernandez & Alani, 2018). The complex world of information presents a challenge in this context: Events are often portrayed with a mixture of true and false information, whether in interviews or witness testimonies (Loftus, 2005; Wixted et al., 2018), in the circulation of rumors (Allport & Postman, 1946; Shin et al., 2018), or in media reports (Lewandowsky et al., 2012). The most widespread fake news articles incorporate a factitious information blend (George et al., 2021). These blends merge accurate details with false information, creating a narrative that appears more plausible to readers.

The effectiveness of such a blend of misinformation is further enhanced by the fact that people are truth-biased (Gilbert et al., 1993; Pantazi et al., 2018). They tend to believe what they read. Therefore, the identification and correct processing of false information is important for creating an accurate representation of described events. At least for media accounts of episodic events, many so-called fact-checkers have emerged that try to validate true statements and identify, mark, and correct false statements accordingly (Ecker et al., 2022). In the simplest form, veracity cues can be used where the falseness of a statement is signaled by a graphical making or a flag that serves as a metacognitive veracity warning signal (Brashier et al., 2021; Grady et al., 2021; Kozyreva et al., 2024). However, these approaches mostly focus on headlines or statements that are factually true or false. While this approach is practical, it overlooks the complexity of narratives that contain a mixture of true and false elements, where statements often vary in veracity and do not always have a factual truth.

Given these limitations, our study focuses on two main research questions: How does labeling information using veracity cues within one coherent event narrative affect information processing? Moreover, how clearly must the differentiation between *true* and *false* veracity cues be made for a correct mental representation of the described event? To explore these questions, we investigate how varying the clarity of veracity cues in coherent fictional event descriptions influences readers' ability to distinguish between information labeled as true or false. With clear veracity cues, we mean how easy it is to differentiate true from false statements based on visual cues (e.g., font color). We analyze how these variations impact content, veracity identification, and confidence in judgment accuracy. We especially focus on coherent fictional event descriptions rather than isolated headlines. We label statements within these descriptions as true or false using visual cues (font color). While these veracity cues may imply some degree of factuality in our context, it is important to note that they do not claim or reflect factual knowledge of real-world events, as all information could be true in everyday situations.

### Accurate mental representation

People want to create an accurate mental representation of described events (Zwaan & Radvansky, 1998). To create this mental representation, the processing of information alongside its veracity includes at least three factors. Firstly, the semantical comprehension of the given information; secondly, the perceptual processing of provided veracity cues, such as color representing the truth value (Gilbert et al., 1993); and thirdly, the validation process of the information veracity. These three processing factors are not necessarily processed one after the other but are highly likely to interact with each other (Richter et al., 2009). Only when all these processes work accurately is a correct mental representation of the event created; otherwise, an inaccurate mental representation is stored and causes memory errors. Factors influencing the creation of an accurate mental representation of events can be the erroneous tagging of information (Gilbert et al., 1993), processing fluency (Oppenheim, 2008), and confidence (Nadarevic & Erdfelder, 2023), which we will discuss in the following in more detail.

### Tagging of false information

People are often truth-biased (Gilbert et al., 1993; Pantazi et al., 2018), meaning they tend to believe and integrate incoming information into their mental representation without validating it. To understand the interaction of comprehension and validation, Gilbert et al. (1990, 1993) proposed two competing models: the Cartesian and the Spinozan models. Both models assume that information processing is divided into two stages and is sensitive to cognitive load. In the Cartesian model, incoming information is stored independently of any true or false tag. A rational analysis of the incoming information happens in a second processing step, where the information receives a true or false tag.

Contrary to that, in the Spinozan model, the incoming information is automatically believed, understood, and accepted as true in the first processing phase. The mental representation is then labeled *false* in the second processing phase. This label is only added to the mental representation when the *veracity tag* of the crucial information is consciously evaluated and perceived. Thus, false information is only rejected when sufficient cognitive resources are available. Results from Gilbert and colleagues (1990, 1993) confirmed that if not enough cognitive resources are available, people tend to be truth-biased and are more likely to remember false statements as true than true statements as false when statements are labeled using veracity cues (e.g., different font colors for true and false statements). However, this pattern of believing false information by default was also shown without a secondary cognitive load task (Pantazi et al., 2018). This is seen as further evidence in favor of the truth bias. These results illustrate how difficult it can be to reject false information, especially when no clear veracity cue is provided to support the validation process.

Research on warning and fact-checking labels uses an external mechanism of tagging information by providing *false* tags or warnings. This approach aims to enhance individuals' ability to discern accurate and inaccurate information (Kozyreva et al., 2024; Pennycook et al., 2020). Pennycook et al. (2020) incorporated a "False" stamp above headlines, demonstrating that accuracy ratings improved when such a cue was present. However, one side effect was an implied truth effect. Untagged headlines (even when factually false) were perceived as more accurate. This observation underscores the importance of ensuring that if veracity cues are used, they should be applied consistently to all provided information.

### Effects of perceptual fluency to decern true from false information

Truth bias can result not only from statements being labeled as true or false but also from processing fluency, defined as the ease of processing information (Oppenheimer, 2008). Two essential factors influence processing fluency, resulting in increased truth judgments: repetition and perceptual fluency. While a large body of research investigating the influence of repetition already exists (for a review, see Dechêne et al., 2010; see also illusory truth effect, e.g., Hassan & Barber, 2021), there is little on perceptual fluency. Alter and Oppenheimer (2009) further differentiate between physical and visual perceptual fluency. The former can be varied by the clarity of the font (e.g., 12-point Times New Roman vs. a small italicized font) and the latter by, for example, the contrast between font color and background.

In an experiment, Reber and Schwarz (1999) varied the difficulty of reading factual true or false statements by changing the contrast between font color and background. Stimuli were presented in black ink on a white background (fluent items) or written in yellow ink on a white background (disfluent items). After only one exposure, participants judged visual perceptual disfluent items on the chance level as true. In contrast, visual perceptual fluent items were judged as true above the chance level independent of their actual truth value.

Importantly, the correlation between visual perceptual fluency and veracity can be learned (Unkelbach, 2007). In a set of experiments, Unkelbach (2007) first replicated Reber and Schwarz's findings by manipulating the visual perceptual fluency of factual true and false statements. Participants judged high-contrast statements more often as true and low-contrast statements more often as false. In a later experiment, they included a learning phase to the paradigm in which visual perceptual fluency and truth were negatively correlated. This resulted in a reversed answer tendency. More recently, Nadarevic et al. (2022) showed that the associations of *green-true* and *red-false* are only present if green and red are present within the same context. Thus, the truth bias effect is sensitive to the ease with which information can be processed and its learned associations.

Research by Alter (2013) provides an important contrasting perspective to these findings by suggesting that disfluency can lead to more careful and thorough processing. Although people generally prefer fluent processing, Alter found that disfluent information processing sometimes results in deeper cognitive processing. For example, participants presented with questions in disfluent fonts were more likely to reconsider their intuitive responses, leading to improved accuracy and better retention of information. This finding suggests that when information is more difficult to process, individuals may engage more deeply, overcoming initial biases and focusing on the meaning of the information. It is important to note that subsequent research has raised questions about the robustness of the disfluency effect, indicating that accuracy improvement may not always be due to disfluency but could also come from the distinctiveness of disfluent materials that capture attention (Eitel et al., 2014; Rummer et al., 2016). Thus, it might also be that true and false information will be better memorized if the discriminability between veracity cues is more difficult and disfluent.

While prior research has explored the role of processing fluency in truth judgments by manipulating the visual ease for individual statements, it has largely overlooked how the clarity of veracity cues between statements affects memory for both content and veracity memory within event narratives. Most existing studies manipulated visual perceptual fluency by altering background and font color in isolated statements or headlines rather than varying the visual perceptual fluency between statements. By exploring how the clarity of this distinction between true and false veracity cues affects memory, we aim to understand better how people process mixed-veracity narratives and whether visual perceptual fluency or disfluency enhances accurate memory formation.

### The role of confidence

While Gilbert and colleagues' research points in the direction of a truth bias based on reduced cognitive resources in the validation processing step, other research suggests that the measurement of uncertainty should also be considered an important variable in this context. Street and Kingstone (2017) found that the truth bias is only present when there is no additional response option *unsure*. Also, Nadarevic and Erdfelder (2013) argue that the truth bias only reflects a guessing bias toward truth, not the actual difference in memory for *true* and *false* veracity cues. Their study showed improved veracity memory for *true* and *false* statements but not for statements of *uncertain* validity status. In a follow-up study, cognitive load during encoding of the veracity tag affected memory for both *false* and *true* trivia statements but only for *true* nonsense statements (Nadarevic & Erdfelder, 2019). Thus, the tagging of the statements influenced memory, partly supporting the Cartesian model. However, additional cognitive load only decreased memory for true nonsense statements, but not for false nonsense statements.

In conclusion, the authors argued for a more complex model in which the tagging is situation-dependent and optional based on the context if the tag is informative. For example, when learning a language, it is more helpful to memorize only true translations, whereas when learning for a multiple-choice test, false information can also be informative in order to exclude incorrect answer options. Mayo (2024) likewise discusses that a truth bias is context-dependent. In a situation where correct information is processed with distrust, accurate information will be rejected faster. This inversely increases the belief in disinformation, challenging the automated truth mechanisms of the Spinozian model. Richter et al. (2009) also argue in favor of a more efficient approach. Results of their study suggest that validation does not happen in a second processing step but instead that individuals validate information simultaneously and fast during comprehension.

One aspect that remains understudied in discussing these models is *propositional confidence*, denoting an individual's confidence in their cognitive judgments, which is crucial for understanding cognitive processes and task performance (Fleming, 2024). Generally, increased confidence in one's judgment is associated with greater accuracy. Integrating this confidence into the framework offers a potential key to resolving factors that neither the Cartesian nor the Spinozan model can fully account for. Examining how veracity cues influence propositional confidence can provide valuable insights, such as why certain information is tagged or confidently believed while other information is dismissed. It bridges gaps in current research and contributes to a more nuanced understanding of veracity cue processing and its relevance for the truth bias.

### The present research

The main research question of this study is how the clarity of veracity cues influences memory and confidence in the context of coherent event narratives, specifically when distinguishing between *true* and *false* cues is difficult. In the present research, the terms *true* and *false* do not imply factual accuracy of the sentence content. These labels are assigned solely based on a visual cue, such as font color, which serves as a veracity cue to indicate the perceived truth status without changing the sentence content. Thus, *true* and *false* function as labels and do not represent factual truth or falsity. To explore this question, we conducted two studies focusing on the role of veracity cues (e.g., font color) in shaping memory. Study 1 examines how veracity cues affect memory for event content (content memory) when the labels true and false are presented through visual cues (i.e., font color), while Study 2 focuses on the memory for the veracity cue of the event content (veracity memory) to investigate whether individuals retain information differently based on its veracity cue. Both studies include confidence in one's accuracy judgments to gain more insights in answering the research question.

The first study (Study 1) aims to investigate the presence and the explicitness of veracity cues on content memory and the role of confidence. Specifically, we examine (i) the truth bias for content memory when veracity cues are present and (ii) the role of confidence. To do this, we vary the clarity of the veracity cues for sentences describing a brief event. Using font color as a cue to signal the sentences' veracity, we adjust the font colors to create either a minimal (low discriminability) or substantial (high discriminability) degree of distinction between *true* and *false* cues. Crucially, the indication of truthfulness is conveyed exclusively through font color rather than the phrasing or content of the sentences.

In line with research on truth bias, we hypothesize that when veracity cues for event sentences are present, participants will remember the content of sentences labeled as true better than the content of sentences labeled as false [H1a]. Information labeled as true can be seen as more relevant for developing a correct situation model of the described event (Zwaan & Radvansky, 1998). This will enhance the processing of true-labeled sentences while decreasing the processing of false-labeled information.

We additionally expect that when the distinction between *true* and *false* cues is minimal (low discriminability), identifying the veracity value requires additional cognitive resources due to the disfluency. This may result in more careful processing of true-labeled and false-labeled information, potentially enhancing memory for both true-labeled and false-labeled content [H1b]. Thus, memory performance will be more evenly distributed between true-labeled and false-labeled information. In contrast, in the high-discriminability condition, participants can easily classify false-labeled information as irrelevant and not memorize the content for an accurate mental representation. This will reduce their content memory for information labeled as false [H1c].

Additionally, we expect that confidence plays a crucial role in this process of recognition of familiar content. We hypothesize that confidence in the given answer will differ between high- and low-discriminability conditions and sentences labeled as true or false [H2a]. If information is presented as true, it is integrated into a correct mental representation and, therefore, better memorized, affecting confidence in the given answer. If information is presented as false, it is less likely to be memorized and reduces confidence during the recognition task. This effect will be stronger in the high-discriminability condition as the identification of the veracity information is easier. We preregistered, in addition, an interaction effect between discriminability, veracity, and confidence on correct recognition, expecting that confidence depending on veracity and discriminability plays a crucial role in correct recognition [H2b].

### Data availability

All data, analysis code, and material for Study 1 and Study 2 are accessible at https://osf.io/e54py/.

## Content memory: Does the presence of veracity cues affect content recognition?

### Methods

**Open science.** The local ethics committee approved the research conducted. We report all measures and exclusions. Study 1 hypotheses, exclusion criteria, dependent variables, and statistical models were preregistered (https://aspredicted.org/WNG_R6Q).

**Sample.** We preregistered to collect a minimum of 177 participants under the assumption of a small effect size (*f* = 0.15, *α* = 0.05, *β* = 0.95). To account for potential data loss, in the end, a total of 224 people participated in the experiment. Participants were recruited through Prolific and received money for their participation. 19 participants failed the color sorting test. Following our preregistration, four participants were identified based on their observation time, which was too short or too long, and they had to be excluded from the subsequent data analyses. Another two participants had to be excluded due to a technical error they reported via Prolific. One participant failed the attention check. The final sample size was *N* = 198 participants (*n*_*female*_ = 93, *n*_*male*_ = 109, *M*_*age*_ = 37.37 ([range_age_ = 18 – 76], *SD*_*age*_ = 12.80). The participants were equally distributed among the three discriminability conditions (66 persons per condition).

**Design.** The study used a mixed design with discriminability (control vs. low vs. high discriminability) as a between-subjects variable and veracity cue (true vs. false) as a within-subjects variable.

**Fig. 1:**
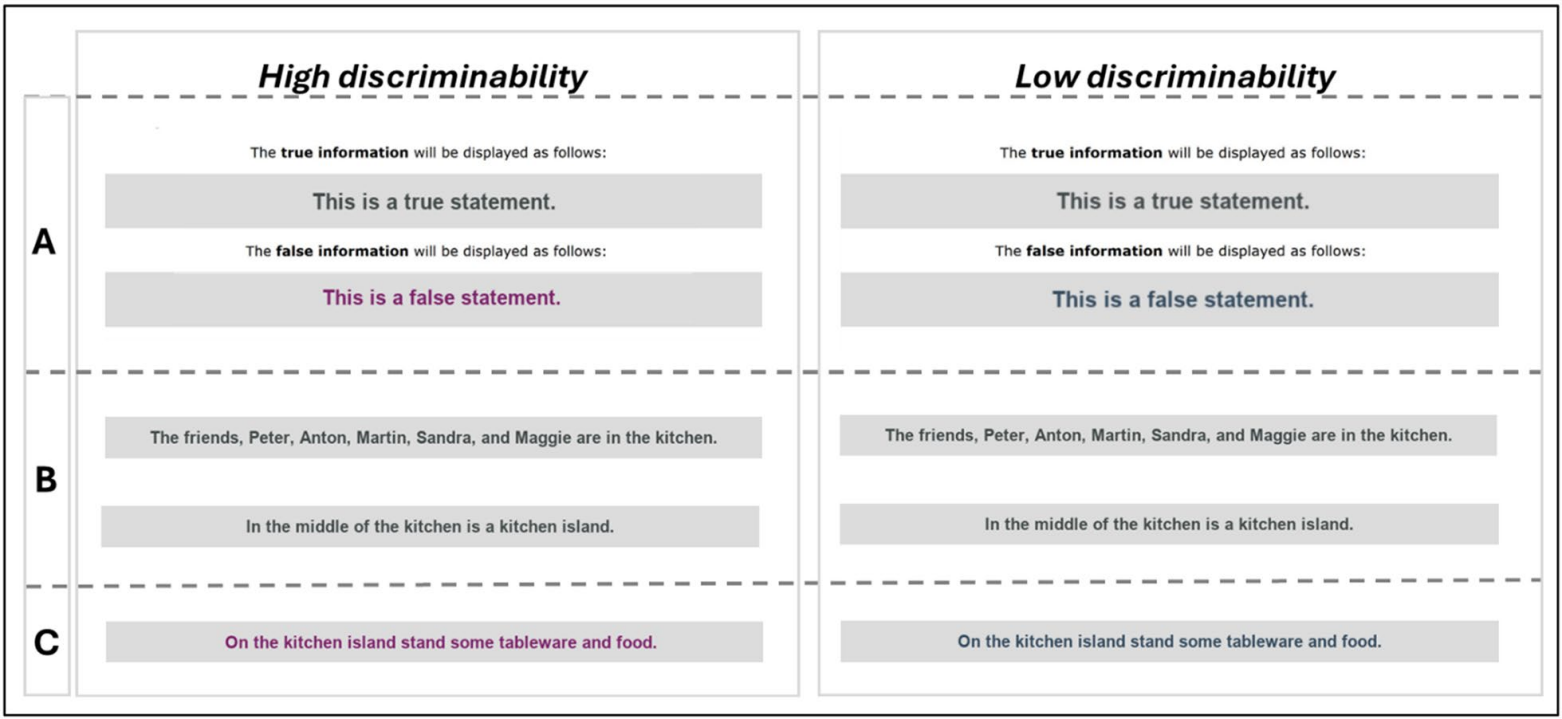
Example stimuli material. The figure depicts in (A) an example of the randomized assignment of the font color as a veracity cue representing true or false in the high and low conditions. (B) shows the two introduction sentences always depicted as *true*, and (C) shows the first target sentence depicted as *false*

**Material.** Participants were presented with one of five event descriptions. Each event description consists of a total of 40 critical sentences. All event descriptions achieved a readability of Flesch > 85 and can be seen as easy to read (Flesch, 1979). The event descriptions were everyday situations, such as friends sitting in the living room and having dinner together (see Appendix A for an example event description). The descriptions were designed so that all sentences were logical and described details of the event (e.g., the appearance of a person, the action of a person, or the object within the event scenery). The basic scenery (room and involved people) was always presented in two introductory sentences written in the font color representing the veracity cue *true*. An additional sentence described the end of the event. This sentence was also always presented in the font color, representing the veracity cue *true*. The two introductory sentences and the end sentences were excluded from the analysis. Of the 40 critical sentences, 30 sentences of the event description were randomly labeled as true, and the remaining 10 sentences as false for each participant. The distinction between *true* and *false* labels was manipulated using font colors on a high or low level of distinguishability (Fig. 1). In the low-discriminability condition, there was a low color distance between the font colors (gray and blue) for the *true* and *false* veracity cues (*∆E* = 8.85), whereas, in the high-discriminability condition, a high color distance was realized between the font colors (gray and purple) for *true* and *false* veracity cues (*∆E* = 30.23). In the control condition, no visual indication was given concerning the veracity of the sentences. The control condition received the sentences in one of the three font colors (blue, gray, or purple). All colors had a high readability score (contrast to background color was higher than 5:1) and were counterbalanced in their veracity representation. That said, we randomized the colors for the different veracity cues. For example, in the high-discriminability condition, gray stood for the veracity label *true* and purple for the veracity label *false* or vice versa.

**Memory test.** All participants were presented with a list of 80 sentences. The list contained all 40 sentences of the event description (old sentences/targets) and 40 new sentences (distractors). For each item, participants were asked to state whether they had read or not read the sentence in the previous event description (yes/no). The 80 sentences depended on the previous read event description. Thus, there are, in total, 200 target items and 200 distractor items.

**Confidence.** For all 80 sentences, participants indicated how certain they were about their answers. The answer options ranged from "50% (I guessed.)" to " 100% (I am certain.)". "50% (I guessed.)" represented the lowest confidence value (1) on the response scale, while "100% (I am certain.)" represented the highest value on the response scale (6).

**Fig. 2:**
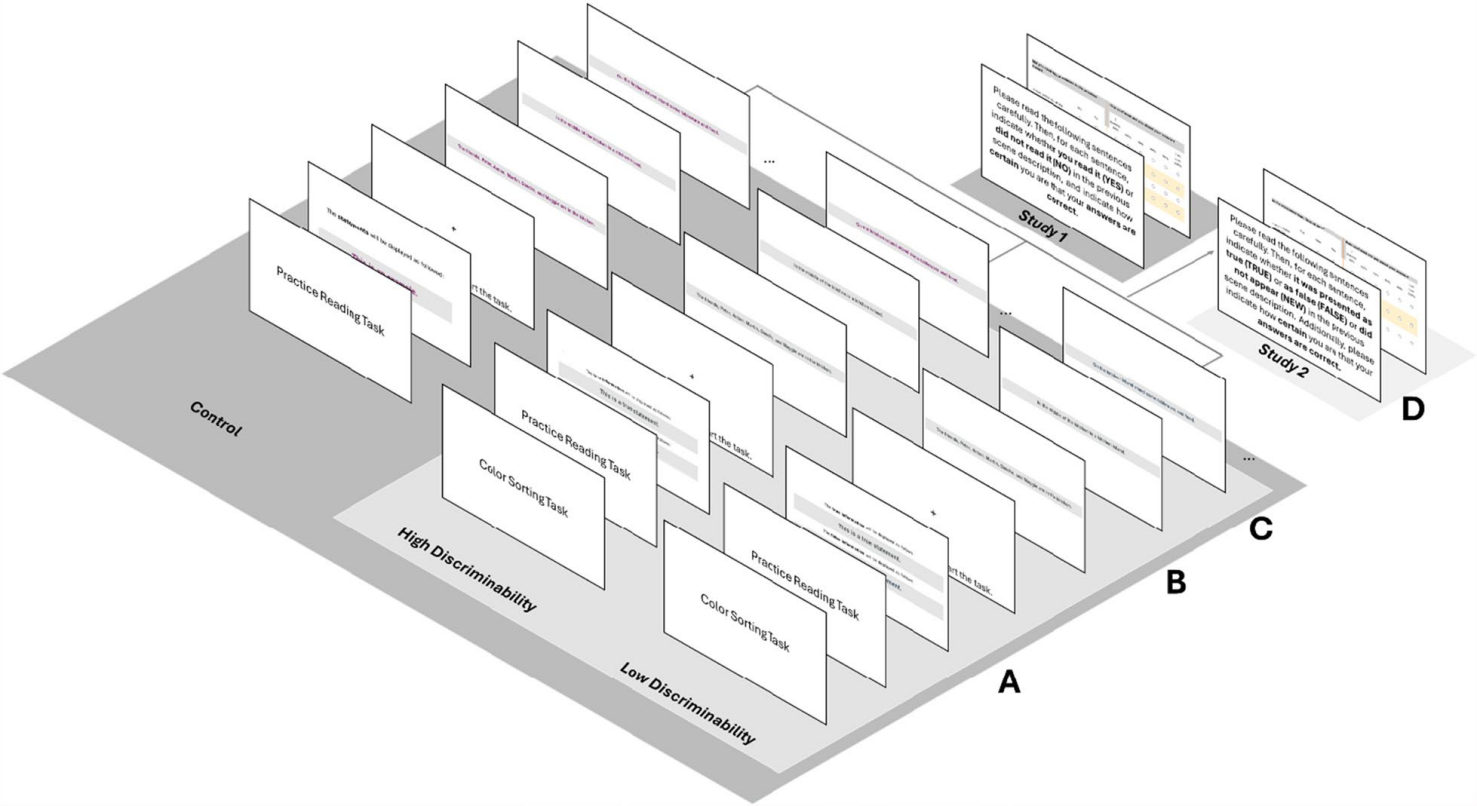
Study Procedure for Study 1 and Study 2. The figure depicts in (A) the start of the reading task with a brief reminder showing the veracity cues in the high and low conditions, while in the control condition, an example statement is shown without any veracity cue. This is followed by two introduction sentences (B) and a first target sentence (C). The encoding phase was similar for Study 1 and Study 2. The retrieval phase (D) differed between the studies (Study 1: content memory; Study 2: veracity memory). Study 1 included the control condition (dark gray surface). Note that the colors representing *true* and *false* were randomized within low-discriminability (blue and gray) and high-discriminability (purple and gray) conditions, while in the control condition, one of the three colors (purple, gray, blue) was randomly used. See also Fig. 1 for examples regarding A, B, and C for the high- and low-discriminability conditions

**Procedure.** Participants were randomly assigned to one of the three discriminability conditions: control, low discriminability, or high discriminability. Participants answered the demographical questions and were then told to read an event description, which they were asked to imagine correctly. Participants in the low- and high-discriminability conditions also learned that the event description contains sentences labeled as true or false in different font colors. The font color representing *true* and *false* was varied between participants. At the same time, however, they were also told that they had to answer questions about all (true-labeled and false-labeled) information afterward. They fulfilled a short practice task to ensure participants could discriminate the different font colors. Participants were informed that there were two different font colors, one representing the veracity value *true* and the other representing *false*. The task was to sort random sentences that were all factually true accordingly into true and false categories based on the sentences' font colors. When the error rate was higher than 25%, participants were asked to repeat the sorting task. If they failed a second time, they could not participate further in the experiment.^1^ The remaining participants and the participants in the control condition continued with the reading task. To familiarize themselves with the reading task, participants first read a short sample event and then read one of the possible five event descriptions (Fig. 2). Directly after, all participants answered the memory test and indicated their confidence in whether their answer was correct. The order of the sentences in the recognition task was randomized. The experiment lasted about 20 min.

### Results

We analyzed content memory and confidence in given answers in two separate mixed models. With these models, we can account for the random influence of participants' individual differences (participant ID) and sentence differences (item ID) while testing for the main effects and interaction of discriminability and sentence veracity. Thus, we included in both models as fixed factors: (i) discriminability (control vs. low vs. high discriminability) and (ii) sentences' veracity cue (true vs. false). Participants' ID and item ID were added as a random intercept to both models. Content memory as a dependent variable was fitted in a generalized mixed-effect model for binomial data since content memory is a binary outcome (correct or incorrect recognition). Confidence as a dependent variable was fitted in a linear mixed-effect model, treating confidence as a continuous variable from low confidence (1) to high confidence (6).

Both resulting models were submitted to a Type III ANOVA. In addition, we fitted both models without the control condition to strengthen our results when there is no comparison between the control condition (without any veracity cue) and the two conditions with veracity cues.

**Descriptive results.** Overall, participants classified 80.14% of all sentences (old and new sentences) correctly. Participants in the control condition correctly identified 83.61%, in the high-discriminability condition 79.23%, and in the low-discriminability condition 77.58% of all sentences, respectively. Thus, overall memory performance was high for all participants. Separating old and new sentences, overall, participants correctly identified 75.88% of the old sentences (control: 79.55%, low: 72.83%, high: 75.25%) and 88.67% of the new sentences (control: 91.74%, low: 87.08%, high: 87.20%).

For subsequent analyses, models were fitted separately for familiar and new sentences. Analyses for the new sentences (distractor) can be found in Appendix C.

**Fig. 3:**
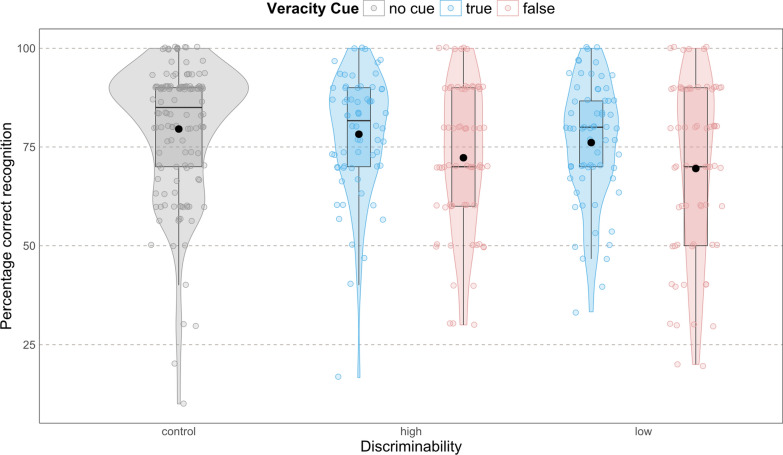
Percentage of Correct Recognition of Event Sentences as a Function of Discriminability and Veracity Cue. In the discriminability condition "control," participants did not see a veracity cue. Black dots represent the mean. Whiskers represent the interquartile range of 95%. *N* = 198

**Content memory.** Beginning with the first analysis of content memory, we were interested in how the presence of the veracity cued affected content memory. If veracity cues are present, we expected participants to remember true sentences better than false ones [H1a]. The model showed an interaction effect between veracity and discriminability, *χ*^*2*^(1) = 7.68, *p* = 0.021. In the high-discriminability condition, the odds of correctly recognizing false compared to true sentences were significantly lower, odds ratio [*OR*] = 0.68 (95% confidence interval [*CI*] = [0.50 – 0.93]), *z* = −2.39, *p* = 0.017. An OR of less than 1 indicates that the event (correctly recognizing false sentences) is less likely to occur in that group compared to the reference group (here, recognizing true sentences). The same effect appeared in the low-discriminability condition, *OR* = 0.67 (95% *CI* = [0.49 – 0.92]),* z* = −2.48, *p* = 0.013. Thus, participants in the low- and high-discriminability conditions remembered sentences presented as *true* better than sentences presented as *false* (Fig. 3 and Table 1). There was no difference in memory performance in the control condition between true and false sentences, *OR* = 0.98 (95% *CI* = [0.70 – 1.37]), *z* = −0.16, *p* = 1.00. This was expected as the control received no veracity cue, and the *true* and *false* labels were randomly assigned for analysis. Thus, when veracity cues were present, sentences labeled as true were remembered better than sentences labeled as false, confirming hypothesis H1a.

**Table 1:** Results of the generalized linear model fitted to Study 1: content memory

Predictor	Odds ratio	β	95% CI	Standardized CI	*z*	*p*
*Fixed effects*						
(Intercept)	4.96	4.96	[3.93 – 6.26]	[3.93 – 6.26]	13.48	< .001
Discriminability [high]	0.89	0.89	[0.65 – 1.21]	[0.65 – 1.21]	−0.75	0.456
Discriminability [low]	0.78	0.78	[0.58 – 1.06]	[0.58 – 1.06]	−1.57	0.117
Veracity cue [false]	1.02	1.02	[0.81 – 1.29]	[0.81 – 1.29]	0.16	0.871
Discriminability [high] x Veracity cue [false]	0.68	0.68	[0.50 – 0.93]	[0.50 – 0.93]	−2.39	0.017
Discriminability [low] x Veracity cue [false]	0.67	0.67	[0.49 – 0.92]	[0.49 – 0.92]	−2.48	0.013
*Random effects*						
*N*_*participant*_	198					
*N*_*item*_	200					
Observations	7,920					

Post hoc simultaneous pairwise comparisons using Tukey's HSD test to adjust for multiple comparisons indicated that high- and low-discriminability conditions were not statistically different in their memory for true, *z* = 0.82, *p* = 0.963, and for false sentences, *z* = 0.75, *p* = 0.975 [H1b and H1c]. However, the low-discriminability condition differed from the control condition for false sentences, *z* = 3.36 *p* = 0.010, but not for true sentences, *z* = 1.57, *p* = 0.621. Post hoc comparison between control and high discriminability did not show a difference in memory performance for false, *z* = 2.61, *p* = 0.094 or true sentences, *z* = 0.75, *p* = 0.976. Thus, against our hypotheses [H1b and H1c], sentences labeled as true or false were not differently processed for content retention depending on how easy or difficult the identification of the veracity cue was.

Fitting the same model, excluding the control condition, resulted in the same effect pattern. In line with the previous model, we did not find an interaction between veracity and discriminability, *χ*^*2*^(1) = 0.01, *p* = 0.933, explaining that the low and high discriminability did not have different memory for true or false sentences [rejecting H1b and H1c]. However, we found again a main effect for veracity, *χ*^*2*^(1) = 11.35, *p* < 0.001, stating that sentences presented as false were generally less often remembered [supporting H1a].

**Confidence.** Turning to the confidence judgment of participants, we expected that confidence would be different for true and false sentences in the discriminability conditions [H2]. The confidence values ranged from 1 to 6, with 1 standing for "50% (I guessed.)" to 6 for "100% (I am certain.)". Descriptively, overall confidence was high, reaching a mean of 4.45 (*SD* = 1.65). Separated by discriminability, confidence was the highest in the control condition with a mean of 4.69 (*SD* = 1.60), followed by high discriminability with a mean of 4.26 (*SD* = 1.60) and low discriminability with a mean of 4.28 (*SD* = 1.72). Before conducting the statistical analyses, the confidence responses of all participants were centered. Fitting the model with confidence as a dependent variable showed no interaction effect between discriminability and veracity cue, *χ*^*2*^(2) = 5.00, *p* = 0.08. Thus, our hypothesis [H2a] that confidence would differ between true and false sentences within discriminability conditions was not supported (Table 2). However, discriminability significantly predicts confidence, *χ*^*2*^(2) = 9.81, *p* = 0.007. Participants in the high-discriminability condition reported lower overall confidence compared to the control condition, *β* = −0.21, 95% *CI* = [−0.62 – −0.06], *p* = 0.018, as did participants in the low-discriminability condition *β* = −0.23, 95% *CI* = [−0.67 – −0.10], *p* = 0.007. There was no difference in confidence judgment between true and false sentences, *χ*^*2*^(1) = 0.05, *p* = 0.826.

**Table 2:** Results of the linear model fitted to Study 1: confidence

Predictor	Estimates	*β*	95% *CI*	Standardized *CI*	*t*	*p*
*Fixed effects*						
(Intercept)	0.24	0.14	0.03 – 0.45	0.02 – 0.27	2.22	0.027
Discriminability [high]	−0.34	−0.21	−0.62 – −0.06	−0.38 – −0.04	−2.36	0.018
Discriminability [low]	−0.39	−0.23	−0.67 – −0.10	−0.40 – −0.06	−2.69	0.007
Veracity cue [false]	0.05	0.03	−0.08 – 0.17	−0.05 – 0.10	0.73	0.465
Discriminability [high] x Veracity cue [false]	0.04	0.02	−0.13 – 0.21	−0.08 – 0.13	0.42	0.676
Discriminability [low] x Veracity cue [false]	−0.15	−0.09	−0.32 – 0.02	−0.19 – 0.01	−1.69	0.091
*Random effects*						
*N*_*participant*_	198					
*N*_*item*_	200					
Observations	7,920					

**Fig. 4:**
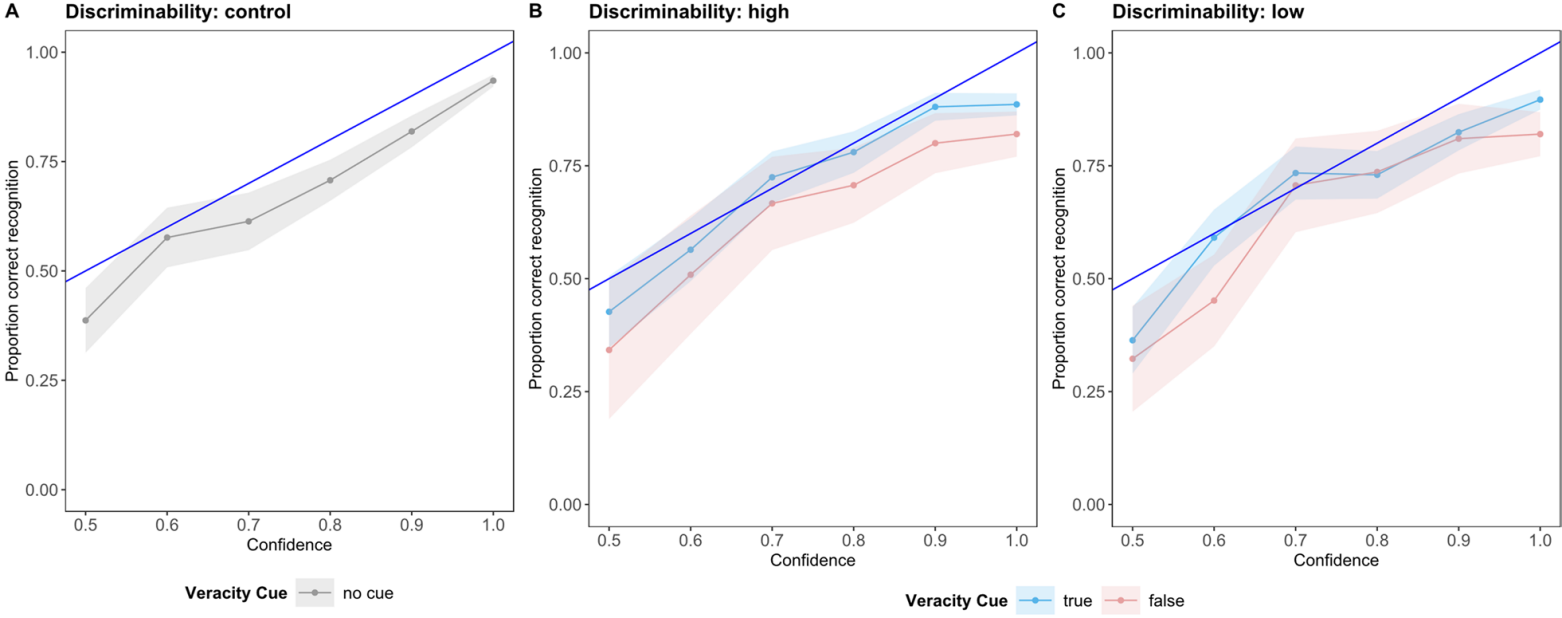
Calibration Plot for Confidence and Correct Recognition separated by Discriminability for Study 1. Confidence was collected on a 6-point Likert scale with answer options "50% (= I guessed.)" to "100% (= I am certain.)". The blue line represents the optimal calibration of confidence and correct content recognition of sentences labeled as true or false. In the control condition, the blue line represents optimal calibration and correct recognition of all sentences, as no veracity cue was provided. Optimal calibration would exist when high confidence predicts high accuracy ("100% (= I am certain.)"), while low confidence predicts low accuracy ("50% (= I guessed.)")

Because we were also interested in the impact of confidence on the given answer, we added confidence as an interaction term to the content memory model. Adding the centered confidence to the model showed that the content memory significantly depends on confidence, *χ*^*2*^(1) = 785.71, *p* < 0.001. The likelihood of a correct recognition was significantly higher when confidence was high. The model showed no interaction effect between discriminability, veracity cue, and confidence, *χ*^*2*^(2) = 0.90, *p* = 0.636. Thus, our hypothesis [H2b] was only partly confirmed. These results are also mirrored in the calibration plots for each discriminability condition (Fig. 4). An optimal calibration would exist when high confidence predicts high accuracy ("100% (= I am certain.)"), while low confidence predicts low accuracy ("50% (= I guessed.)"). The plots show that confidence was well-calibrated with content memory accuracy across all conditions and for both true-labeled and false-labeled sentences in the high- and low-discriminability conditions. The table for the complete model can be found in Appendix B.

### Discussion

Firstly, we will discuss the results of the truth bias effect on content memory when veracity cues are present. In line with our hypotheses, we found that the additional veracity cue influenced the recognition of the content. Most importantly, we found an interaction effect between veracity cue and discriminability. In the control condition, all sentences were recognized equally well. Further, participants in the two discriminability conditions recognized sentences labeled as true more often than sentences labeled as false. Therefore, our results indicate a directed forgetting of false-labeled information (Kimball & Bjork, 2002). Even though the participants were not told to forget the false-labeled information, the intentional forgetting of the information tagged as false is of great importance for the goal of creating an accurate mental representation (Zwaan & Radvansky, 1998). In the control condition, all information was relevant and could not be selectively stored. In the discriminability conditions, the presented veracity cue guided how the information is processed and stored. However, although there was a difference in visual perceptual fluency between the high- and low-discriminability conditions, we did not find any difference in their recognition. Consequently, the level of discriminability does not affect the tagging process or reduce content memory even more. Thus, the study only partly confirmed our hypotheses.

A post hoc test showed that the low-discriminability and control condition differed in their memory performance for false-labeled sentences. Neither the low- and high-discriminability conditions nor the high-discriminability and control conditions differed in their recognition of false-labeled sentences. This result could indicate that the presence of veracity cues and the more difficult discriminability require a higher cognitive effort at the expense of general content memory performance. However, these results should be viewed cautiously, considering they follow a post hoc analysis and that there was neither a difference between high-discriminability and control conditions nor between high- and low-discriminability conditions.

Secondly, turning to the results for the confidence measure, we found that the presence of veracity cues reduced confidence, which is in line with our prediction. Interestingly, we did not find an effect of veracity. Although content memory was reduced for sentences labeled as false, confidence was equally low for sentences labeled as true and false. This result contradicts our previously stated hypothesis. One explanation could be that it is not the individual veracity cue that affects confidence in the response but the additional challenge of accurately labeling the information to create a correct mental representation that affects participants' confidence in their judgment and their confidence in their overall memory performance.

To summarize, the first study indicates that veracity cues impact both content memory and confidence for event descriptions. The veracity cues alongside the sentences are processed in a way that reduces the likelihood of false-labeled information being included in content memory. This suggests that participants could resist information labeled as false, resulting in a more accurate representation of the situation model.

For our second study, it was essential that the memory performance for sentences labeled as true and false between the two discriminability conditions did not differ in Study 1. As an important assumption for Study 2, we expect that people in both discriminability conditions perceive all content information equally and that recognition is on the same level. In the second study, our focus is on veracity memory. Here, we investigate whether the difficulty in identifying the accurate veracity cue affects participants' veracity memory for the sentences of the event description.

## Veracity memory: does discriminability of veracity cues affect truth bias?

Building upon the results of Study 1, Study 2 shifts the focus from content memory to veracity memory. This shift provides deeper insight into how information—whether labeled as *true* or *false*—is stored and whether individuals can access its veracity after retention. We investigate the extent to which the difficulty in discriminating veracity cues affects veracity memory (i.e., memory for the truth value of sentences). Thus, we aim to (i) investigate the truth bias in veracity memory when veracity cues are challenging to identify and (ii) examine the role of confidence in this context.

We hypothesize that veracity memory will be reduced if the discriminability between true and false veracity cues is low [H3]. Consequently, if the truth value classification gets more difficult, participants' mental representation will become less accurate. Thus, the truth bias effect will become stronger. A low level of discriminative veracity cues can lead to a higher level of cognitive load, and therefore, the validation processing might suffer. Thus, low discriminability results in less correctly classified sentences [H3a]. In comparison, if the veracity cue is easy to process, then veracity memory will be higher, and participants will classify true-labeled and false-labeled sentences more often correctly [H3b]. Concerning the truth bias, the lack of accurate veracity processing may explain why people are more likely to remember false-labeled sentences as true (Pantazi et al., 2018).

We also hypothesized that confidence will differ between the levels of discriminability and veracity^2^ [H4]. When the discriminability between true and false sentences is low, it is more difficult to identify the correct veracity value, which increases uncertainty in return [H4a]. This effect will be stronger for false sentences because these sentences are less often memorized. Thus, participants in the low-discriminability condition will have the lowest confidence for false sentences [H4b]. We were additionally interested in whether confidence can predict correct classification. We hypothesized that confidence would be higher for correctly classified than for falsely classified sentences [H4c].

In summary, Study 2 tests whether the memory of the veracity of sentences is affected by the discriminability of this veracity cue. We expect participants in the high-discriminability condition to have better memory for the truth value than participants in the low-discriminability condition. Additionally, we expect confidence to be higher in the high-discriminability condition than in the low-discriminability condition.

### Methods

**Open science.** This study was approved by the local ethics committee and preregistered on AsPredicted.org (https://aspredicted.org/XGX_VHS). For data analyses, we will only report the analyses based on the responses, which we preregistered next to an analysis based on Pantazi et al. (2018).^3^

**Sample.** Based on the results of Study 1, we conducted a power simulation with a mixed-effect model, including an interaction effect for sentences' veracity and discriminability (*β* = 0.3). To achieve a power of 0.80, we planned to achieve a minimum sample size of 220 participants. In the end, 300 participants were recruited through Prolific and received money for their participation. In total, 52 participants had to be excluded from subsequent data analysis due to failing the color sorting test (*N* = 43) and observation time (*N* = 8). Additionally, one participant failed the attention check. The final sample consisted of *N* = 248 participants (*n*_*female*_ = 119, *n*_*male*_ = 126, *M*_*age*_ = 38.08 [range_age_ = 18–77], *SD*_*age*_ = 11.77), with 123 participants in the low-discriminability condition and 125 participants in the high-discriminability condition.

**Design.** The study used a mixed design with discriminability (low vs. high discriminability) as the between-subjects variable and veracity (true vs. false) as the within-subjects variable.

**Material.** The material was identical to that of Study 1.

**Veracity memory task.** The same lists of sentences were used as in Study 1. However, for each item, participants were asked to state whether the sentence was presented as true or false in the previous event description or whether it was a new sentence.

**Confidence.** Similar to Study 1, we asked participants how certain they were about their answers. They could select on a 6-point Likert scale from "I guessed. (50%)" to "I am certain. (100%)".

**Procedure.** We followed the same procedure as in Study 1. The only difference was that participants were randomly assigned to two discriminability conditions instead of three, and, in the end, they fulfilled the veracity memory task with the confidence rating.

### Results

The dependent variable, veracity memory, was fitted using a generalized mixed-effects model for binomial data. Additionally, a linear mixed-effects model was fitted for confidence, the second dependent variable. We incorporated two fixed factors in both models: (i) discriminability (low vs. high discriminability) and (ii) veracity (true vs. false). Participants could correctly identify each sentence as true or false or misidentify it as new. Additionally, true sentences could be confused as false and false sentences as true. Thus, responses can be sorted into three classifications: correct, confused, and misidentified. Therefore, we also fitted separate models for the incorrect responses, which were either confused or misidentified. Thus, every item response was transferred to 1 based on their answer in that classification. For example, if a sentence had the veracity *false* and the participant responded *true*, then on the classification level *confused*, the response was labeled as 1. In contrast, on the classification levels *correct* or *misidentified*, it was labeled as 0.

To account for individual variations, we included participants' and item IDs as random intercepts in the models. All resultant models (for veracity memory and confidence) were submitted to a Type III ANOVA for analysis.

**Descriptive results.** Overall, participants correctly identified 51.18% of the familiar sentences (low: 49.34%, high: 53.00%) and 63.98% of the distractor sentences (low: 64.73%, high: 63.24%). In the low-discriminability condition, participants confused 25.20% of the familiar sentences and misidentified 17.50% as new sentences. In the high-discriminability condition, participants confused 24.00% of the familiar sentences and misidentified 15.50%.

For subsequent analyses, models were fitted separately for target and distractor sentences. An analysis of the distractor sentences can be found in Appendix D.

**Fig. 5:**
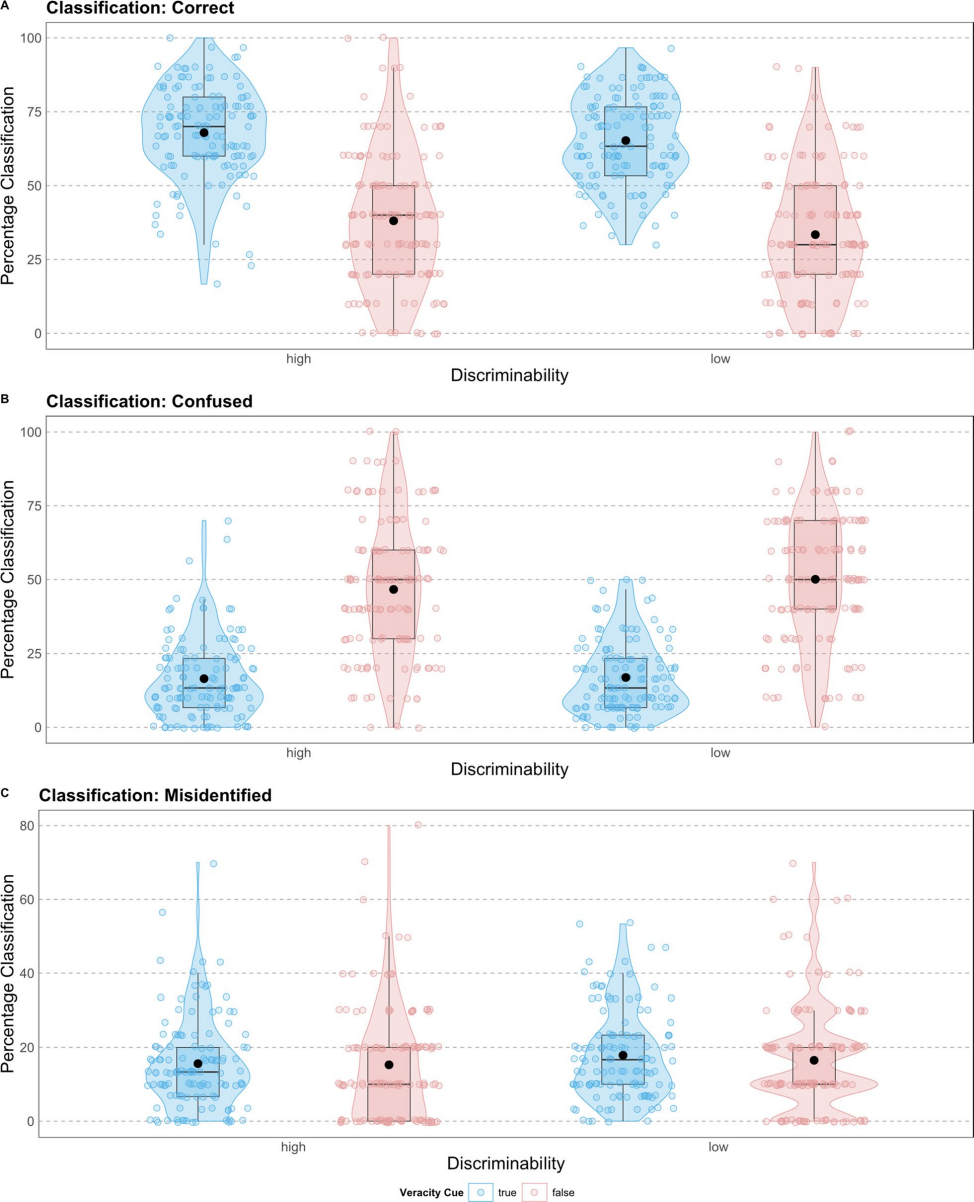
Percentage of given responses (classified as correct, confused, and misidentified) for Study 2: source memory as a function of discriminability and veracity cue. The plots (A), (B), and (C) represent the descriptive data for the responses given by the participants. Black dots represent the mean. *N* = 248

**Veracity memory.** As a first analysis, in line with hypothesis H3, we tested how veracity memory is affected by discriminability and veracity. For the model parameter, see Table 3. The model showed a significant main effect of veracity, *χ*^*2*^(1) = 350.31, *p* < 0.001. False sentences were significantly less often correctly identified than true sentences, *OR* = 0.26, 95% *CI* = [0.23 - 0.30], *p* < 0.001. This supports hypothesis H3a, which states that participants were more likely to remember the veracity value of true sentences correctly than false ones (Fig. 5).

**Table 3:** Results of the generalized linear model fitted to Study 2: veracity memory

Predictor	Odds ratio	*β*	95% *CI*	Standardized *CI*	*z*	*p*
*Fixed effects*						
(Intercept)	2.25	2.25	[1.99 – 2.55]	[1.99 – 2.55]	12.89	< .001
Discriminability [low]	0.88	0.88	[0.74 – 1.03]	[0.74 – 1.03]	−1.59	0.112
Veracity cue[false]	0.26	0.26	[0.23 – 0.30]	[0.23 – 0.30]	−18.72	< .001
Discriminability [low] x Veracity cue[false]	0.93	0.93	[0.76 – 1.13]	[0.76 – 1.13]	−0.74	0.458
*Random effects*						
*N*_*participant*_	248					
*N*_*item*_	200					
Observations	9,920

Importantly, we did not find an interaction between discriminability and veracity, *χ*^*2*^(1) = 0.55 *p* = 0.458. The correct veracity memory for true and false sentences did not differ between the low- and high-discriminability conditions. Thus, contrary to H3b, the ability to identify the correct veracity value was not significantly influenced by the degree of discriminability between the veracity cues.

Based on the truth bias assumption, we did an exploratory analysis only looking at the sentences that were not correctly classified. These sentences could be confused (true-labeled as false or false-labeled as true identified) or misidentified (true/false as new). The model based on answers classified as confused showed a strong veracity effect, *χ*^*2*^(1) = 115.72, *p* < 0.001. False-labeled sentences were more often confused as true than true-labeled sentences as false, *OR* = 3.68, 95% *CI* = [2.90, 4.67], *p* < 0.001. The results suggest a strong truth bias effect when the sentences were incorrectly classified. Additionally, answers classified as misidentified differed between true-labeled and false-labeled sentences, *χ*^*2*^(1) = 115.70, *p* < 0.001. False sentences were less often misidentified compared to true sentences, *OR* = 0.27, 95% *CI* = [0.21, 0.34], *p* < 0.001. These effects were independent of the discriminability condition in both models (Fig. 5).

We could also show that the classification (correct, confused, or misidentified) significantly differed based on the discriminability, *χ*^*2*^(2) = 11.59, *p* = 0.003. Participants in the low-discriminability condition more often misidentified sentences, *χ*^*2*^(1) = 7.05, *p* = 0.008, and less often correctly identified sentences compared to the high discriminability condition, *χ*^*2*^(1) = 10.11, *p* = 0.001. Both conditions equally often confused sentences' veracity, *χ*^*2*^(1) = 1.75, *p* = 0.186. Thus, the increased difficulty to discriminate between true-labeled and false-labeled sentences affected the general memory performance but did not result in a higher confusion rate of the veracity value.

**Confidence.** We were interested in whether the confidence between the discriminability conditions differed based on the veracity of the sentences. Overall confidence reached a mean of 3.77 (*SD* = 1.73). Separated by discriminability, confidence was higher in the high-discriminability condition (*M* = 3.88, *SD* = 1.72) than in the low-discriminability condition (*M* = 3.66, *SD* = 1.73). For true sentences, confidence was slightly higher in the high-discriminability condition (*M* = 3.87, *SD* = 1.72) than in the low-discriminability condition (*M* = 3.65, *SD* = 1.73). For false sentences, confidence was also higher in the high-discriminability condition (*M* = 3.92, *SD* = 1.73) than in the low-discriminability condition (*M* = 3.71, *SD* = 1.74).

Given the minimal descriptive differences, we did not find a significant two-way interaction between veracity and discriminability, *χ*^*2*^(1) = 0.122, *p* = 0.726. Thus, there was no difference in confidence between discriminability and veracity, which is contrary to what we hypothesized in H4. Analyzing the responses based on their classification (correct, confused, misidentified) showed that confidence was dependent on veracity for confused responses, *χ*^*2*^(1) = 87.79, *p* < 0.001. When participants confused false sentences, they were more confident in their answer than when they confused true sentences, *β* = 0.72, 95% *CI* = [0.57, 0.87], *p* < 0.001. Thus, indicating confidence value depends to a certain degree on the veracity value of sentences and results in disproportional high confidence for false sentences confused as true. For correct responses, *χ*^*2*^(1) = 1.35, *p* = 0.244, and for misidentified responses,* χ*^*2*^(1) = 0.29, *p* = 0.593, we could not find a veracity effect.

**Fig. 6:**
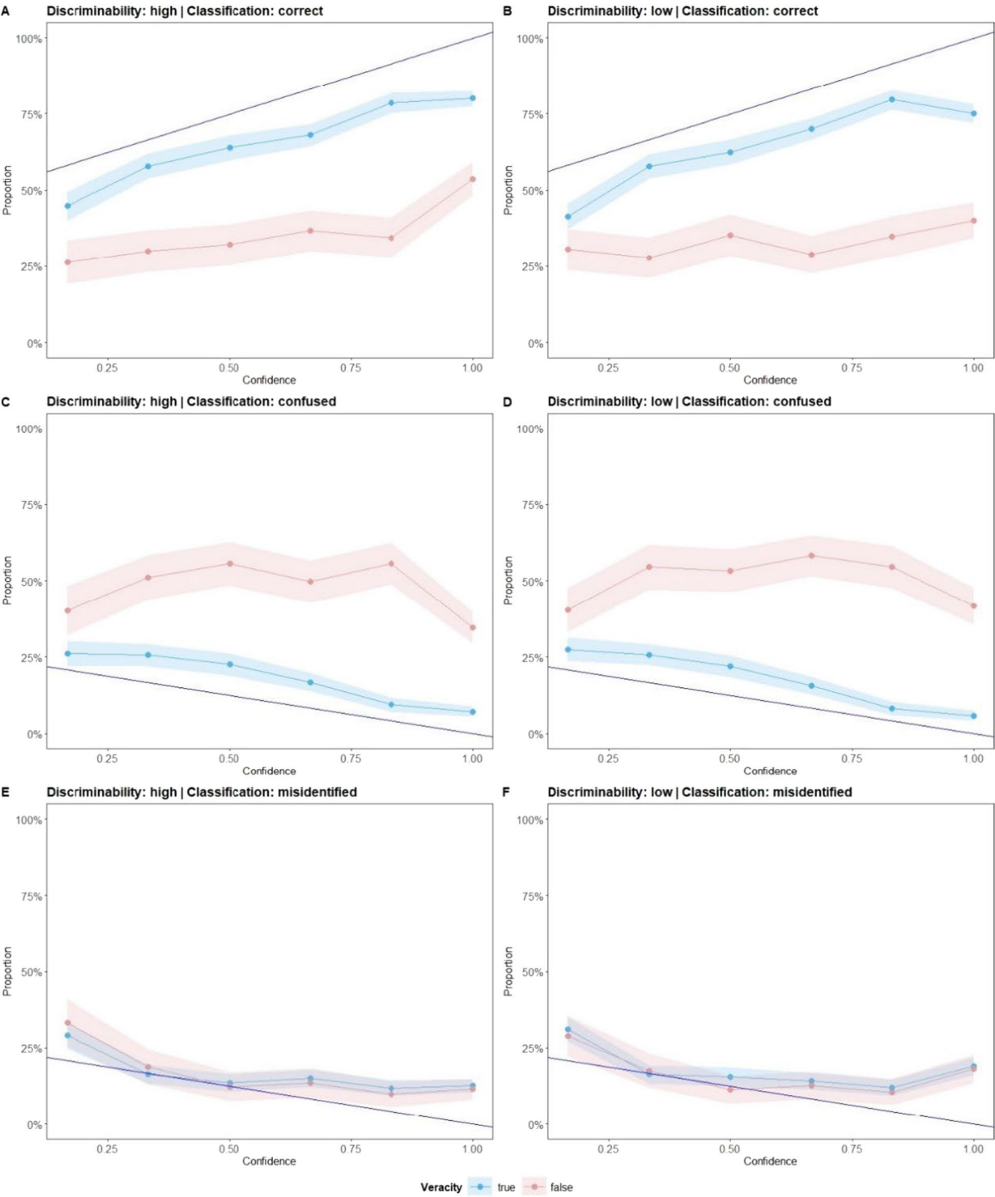
Calibration plot for confidence and responses (classified as correct, confused, and misidentified) separated by discriminability and veracity for Study 2. Confidence was collected on a scale from "50% (= I guessed.)" to "100% (= I am certain.)" The blue line represents the optimal calibration of confidence and response classification of true-labeled and false-labeled sentences. For the responses classified as confused or misidentified, the slope is negative as with higher confidence, fewer errors should be made

We also were interested in how confidence can predict correct classification [H4c]. For that, we included confidence in the veracity memory model as an additional fixed effect and submitted the whole model to a Type III ANOVA. Figure 6 shows how confidence differed based on the proportion of responses separated by classification. We expect that with higher confidence, the proportion of correct responses increases, while for confused or misidentified veracity memory, the proportion decreases with higher confidence. The results showed a significant main effect of confidence, *χ*^*2*^(1) = 192.18, *p* < 0.001, indicating that higher confidence was associated with a higher likelihood of correct veracity memory. This is in line with our hypothesis H4c. Interestingly, there was a significant interaction between veracity and confidence, *χ*^*2*^(1) = 4.27, *p* = 0.039, showing that confidence was a stronger predictor of correct veracity memory for true sentences than for false sentences. Thus, confidence was better calibrated for true sentences. This supports our previous finding of increased confidence for false sentences that were confused as true.

The three-way interaction between discriminability, veracity, and confidence was also significant, *χ*^*2*^(1) = 5.55, *p* = 0.019. Overall, participants reported higher confidence in the high-discriminability condition compared to the low-discriminability condition, with this effect being most evident for false sentences. Specifically, participants in the low-discriminability condition showed significantly lower confidence in their correct responses for false sentences compared to those in the high discriminability condition, *z* = 15.94, *p* < 0.0001. This suggests that when distinguishing true from false sentences is difficult, participants' confidence, especially when recalling false sentences, barely predicts correct recognition. For true sentences, confidence levels did not differ significantly between the discriminability conditions.

### Discussion

In line with our hypotheses, veracity memory for event descriptions was higher for true sentences than for false ones, as participants were more likely to classify true sentences as true compared to false sentences as false. This supports the assumption that true sentences are better integrated into the mental event model for an accurate representation, which increases memory (Zwaan & Radvansky, 1998). However, we did not find an interaction between discriminability and veracity. Contrary to hypothesis H3b, the ability to identify the correct veracity value was not significantly influenced by the clarity of the veracity cue. This suggests that while the presence of veracity cues especially affects false-labeled sentences, it does that independently of the difficulty of the discrimination task.

The exploratory analysis showed that when sentences were incorrectly classified, false-labeled sentences were more often confused as true compared to true-labeled sentences as false. This favors the truth bias assumption (Pantazi et al., 2018). The higher confusion of false-labeled sentences as being true supports the theoretical approach of the Spinozian model, according to which only false sentences receive a tag; otherwise, sentences are believed to be true by default (Gilbert et al., 1993). The results indicate that the false-labeled sentences lost their false tag, or the false tag was not memorized in the first place. The missing false tag but integrating the content into the mental representation could serve as a cue for classifying false-labeled sentences as true.

Interestingly, participants in the low-discriminability condition overall misidentified sentences (independent of veracity cue) more often than those in the high-discriminability condition, while the confusion rate was at a similar level across both conditions. Thus, the difficulty of the discrimination task affected veracity memory for both true-labeled and false-labeled sentences. The easier it was to process the cue about the veracity value, the higher the probability was that the veracity value was accurately remembered, and a complete mental model, including the veracity tags for each sentence, was created. Thus, the increased difficulty in discriminating between true and false sentences affected general memory performance but did not increase confusion rates. This finding is more in favor of the Cartesian model and contradicts the approach of the Spinozian model, which assumes a higher confusion rate and lower memory performance for false sentences only.

In line with Study 1, confidence significantly depended on veracity. Participants were more confident when their answer was correct than when it was confused or misidentified. The high confidence for correct classified sentences can be based on the accurate mental model of the event, including the false tags. For true sentences, confidence was also higher compared to false sentences. However, we found a significant difference between true and false sentences once participants confused sentences. When participants confused false-labeled sentences as true, their confidence was about the same level as the correctly classified sentences. This finding highlights that judgment in the answer was not well-calibrated as confidence disproportionately increased for false sentences confused as true. For true-labeled sentences confused as false, confidence was better calibrated. Additionally, confidence was lower if it was more difficult to identify the correct veracity cue.

To conclude, our results of Study 2 show that reading an event description presented with additional veracity cues affects memory. Firstly, false-labeled information is more likely to be confused as true. Secondly, the more demanding the discriminability task is, the more likely it is that less veracity information is remembered independent of the cue (*true* or *false*). While the Spinozian model accounts for the higher confusion rate of false-labeled sentences, the similar forgetting and correct identification rate of true-labeled *and* false-labeled sentences aligns more with the Cartesian model. Thus, processing event information with *true* and *false* veracity cues cannot completely be explained by the Spinozian nor by the Cartesian model.

## General discussion

Central to our research was the question of how individuals process a mix of true-labeled and false-labeled information within coherent event descriptions and how the clarity of these veracity cues influences the accuracy of memory for the content and the veracity value, as well as the effect on propositional confidence. With this, we aimed to contribute to the theoretical understanding of the truth bias and further enlighten the discussion around the Spinozian and Cartesian models. Our results offer additional insights into how people process a blend of true and false information often encountered in media and narratives.

In general, we have four key findings: First, the presence of true and false labels (veracity cues in the high- and low-discriminability condition) within a coherent event narrative reduces memory for details of the event compared to when no veracity cue is present (Study 1). Second, people are more uncertain about their content memory of the event when veracity cues need to be processed (low- and high-discriminability condition compared to control condition; Study 1). Third, the veracity cues for details labeled as true of the event are better remembered, while details labeled as false are more often confused as true (in low- and high-discriminability conditions; Study 2). Fourth, people are more confident in identifying the veracity value of details labeled as true but also disproportionally confident when misjudging false-labeled details as true (in low- and high-discriminability conditions; Study 2). These results hold across the different levels of discriminability between the veracity cues.

### Truth bias and processing models

The main research question of this study was how the clarity of veracity cues influences memory within coherent event narratives, specifically when distinguishing between true and false veracity cues is difficult. To address this, we looked at truth bias in content and veracity memory when veracity cues are present. Our findings that true-labeled information is better recalled and false-labeled information is more often confused as true align with previous research, suggesting that truth bias results from automatic acceptance of incoming information (Gilbert et al., 1993; Pantazi et al., 2018). However, neither processing fluency (Reber & Schwarz, 1999) nor processing disfluency (Alter, 2013) seem to play a major role in mitigating the truth bias. This suggests that the tendency to accept information as true is not strongly influenced by how easily it is processed. Only in the second study was memory independent of the veracity cue reduced in the low-discriminability condition as they misidentified more sentences as new. That said, the found effects appear to persist even when the clarity of the veracity cues varies (Eitel et al., 2014; Rummer et al., 2016).

Considering our findings for the memory of coherent event descriptions in the context of the Spinozian or the Cartesian model, a more nuanced evaluation is evident. Our results are consistent with the Spinozian model so far as more often false information tends to be mistaken as true than vice versa (Gilbert et al., 1993; Marsh & Fazio, 2006; Pantazi et al., 2018), which is also reflected in an overestimation of confidence for these false judgments. This confirms the Spinozian model, according to which information is automatically believed and accepted as true.

Conversely, the Cartesian model suggests that all information is initially stored without any veracity tag and that the tags for true and false are only added after a second analysis. This model implies that additional cognitive resources are needed for the accurate labeling of information as both true and false. Our results show that high cognitive demands, such as reduced discriminability of veracity tags, affected overall memory accuracy and confidence. Memory and confidence were reduced not only for false but also for true sentences independent of the discriminability. This aligns with the idea that information is equally processed and that cognitive resources play a crucial role in the accurate processing and labeling of both veracity values, as proposed by the Cartesian model.

Thus, our findings based on event descriptions diverge from the strict predictions of both models. This result indicates that a more comprehensive, situation-dependent model may be necessary to fully capture the complexities of veracity processing and memory accuracy in the light of event descriptions (Nadarevic & Erdfelder, 2019).

### Confidence as a bridging factor

Our research at present gives one potential explanation with the help of confidence, which could bridge the gap between the Spinozian and Cartesian models in the context of event descriptions. The inclusion of confidence provides an additional layer of understanding beyond the Spinozian and Cartesian models, which do not address confidence in their framework. In our study, confidence appeared to be a subjective indicator of how well participants believed they processed content and veracity. This suggests that propositional confidence acts both as a reflection of memory outcomes and as a clarity of veracity cues.

The heightened confidence observed when participants confuse false-labeled information as true aligns with the Spinozian notion of automatic belief. Conversely, participants' reduced confidence when veracity cues were present and the same level of confidence judgments regardless of the discriminability suggests a potential issue in the accurate labeling process for true and false information. This resonates with the Cartesian idea that enough cognitive resources are needed for accurate analysis and tagging of both information. When this process is compromised, individuals may express lower confidence independent of the veracity. Their confidence rating introduces a subjective evaluation of the processing of the content information as well as the veracity cues (Fleming, 2024). When there is a need to distinguish between true and false cues while processing event information, overall confidence may decrease. This increased uncertainty about determining the veracity of a single information might increase doubt in the judgment process itself and is not necessarily a consequence of the truth bias.

The subjective evaluation of information processing, which is reflected in different levels of confidence depending on the situation, could be influenced by the accuracy and memory of the mental models created. That is, the creation of an event model may affect the labeling process in the context of processing event information. According to the understanding of building situation models aiming at accuracy (Zwaan & Radvansky, 1998), uncertainty about a label while integrating the information into the situation model could lead to higher confidence that false information must be true. At the same time, higher uncertainty about a piece of information could serve as an indicator that the information has not been integrated into the situation model and is thus declared unfamiliar. In other words, high confidence is associated with information labeled as true, and low confidence with unfamiliar or false information.

### Practical implications

This study also offers practical insights into processing true and false information. In everyday contexts such as news consumption or social media, true and false information are often blended. Our findings emphasize that veracity cues, such as labels used by fact-checkers, can influence memory accuracy and confidence in accurately judging both true and false information. In the context of coherent event narratives, only a small proportion of information labeled as false is directly forgotten. Most of the information is still remembered and integrated into the mental representation, for which the additional false tag is often forgotten and thus assumed to be true. Taken together, a simple color label does not seem to be a sufficient intervention to successfully counteract the truth bias or enhance direct forgetting. Stronger interventions such as crossing out or graying out false statements within one narrative could be more promising in order to signal more strongly that these sentences are false and could be skipped. However, it is important to note that the pure presence of labels can also reduce accurate memory for true information and make people less certain about what they know. This suggests that labeling may not be an effective way to protect people against false information, as it can introduce cognitive load and reduce both memory accuracy and confidence for all information. More robust interventions may be needed to mitigate the influence of false information without negatively affecting the recall of true information.

### Limitations and future directions

However, our research also has some limitations. Against our assumption, the level of discriminability of the veracity cues did not affect the overall recognition performance compared to the control condition. Thus, the additional effort to identify the veracity cue to validate the information might not be as important as expected. There are two possible explanations for that. Firstly, veracity cues can serve as a cue for remembering content. In line with research on sourcing strategies, comprehension and memory increase when people mentally represent source information (Braasch & Kessler, 2021). Thus, although the presence of veracity cues increases the overall bits of information to process, the more thorough processing of each content with its veracity information increases overall memory performance. This might become even stronger when additional cognitive resources are needed when true and false veracity cues are difficult to distinguish. However, we found a decreased content and veracity memory for false information, which would speak against the deeper processing of false information but for direct forgetting (Kimball & Bjork, 2002).

Secondly, we encouraged participants to represent the event mentally. This instruction method of mental imagery increases learning and memory (Leahy & Sweller, 2008). The mental imagery method, however, might also explain why participants in our studies were less likely to remember false information. Although we emphasized that they would be tested on all given information, correctly creating a mental image of the event would focus on true information while dropping false information (Zwaan & Radvansky, 1998). Thus, the false information already was less likely to be deeply processed. However, participants showed recognition performance for the content way above the chance level for false sentences. In addition, overall memory performance did not differ between the discriminability conditions in our first study. Therefore, more research is needed to understand the interaction and processing of veracity information and the mental visualization of events.

Another limitation of our research concerns the interpretation of veracity memory. Our veracity memory measure does not control for guessing processes. Thus, it captures both memory and guessing. With the confidence judgment and the additional model where we included our confidence measure, we account for guessing influences and show that confidence in one's judgment predicts accurate veracity memory. Nevertheless, future research should consider refining this measurement to distinguish between these components more effectively.

The present research has another limitation regarding how much it accurately represents real-life situations. In this study, the manipulation of veracity information was done by altering font color within an everyday event description. However, in social media, strategies such as "Disputed" or "Rated false" tags are commonly utilized to label entire news articles without going into details in the article (Clayton et al., 2020). Additionally, exposure to true and false information in real-life examples is likely multimodal (e.g., audio, picture, video). Furthermore, how the information is processed may depend not only on the labeling but also on the individuals' attitudes (Mayo, 2024). However, in our study, we did not attempt to place participants in a particular trust or distrust mindset, nor did we use news articles or added images. For future research, it would be interesting to systematically examine the effects of presenting true and false information by varying the discriminability in a multimodal manner, placing people in different mindsets, or using real news articles with a blend of true and false information.

Closely related, future research could also consider the role of veracity cues in processing information that aligns or conflicts with prior knowledge. While our study used fictional event descriptions with little prior knowledge implications, the findings suggest that veracity cues could either reinforce or challenge pre-existing beliefs, depending on their clarity and consistency with those beliefs. When prior knowledge supports the veracity cue (e.g., a "true" label on a factually accurate statement), memory and confidence may be enhanced. Conversely, when the veracity cue contradicts prior knowledge (e.g., a *false* label on a factually accurate statement), it might prompt deeper cognitive engagement to reconcile the conflict. Future research could extend our findings by investigating how the veracity cue interacts with prior knowledge and belief by using information blends in complex narratives with polarizing contexts, such as climate change or political discourse.

## Conclusions

Exposure to veracity cues significantly impacts memory and confidence judgment, leading individuals to believe false information as true. The present research extends previous findings on the truth bias effects. It provides information about the relevance of confidence for event descriptions, which consists of a blend of information labeled as true or false. The presence of veracity cues reduced memory for false-labeled information and decreased the accuracy of veracity classification, especially when the discriminability of the veracity cue was more challenging. Confidence in judgment plays a relevant role when correctly identifying content and veracity value. However, a major issue arises when false information is mistakenly believed to be true, leading to high confidence. These findings can help better understand the processing of false information and provide insights for memory accuracy in the presence of veracity cues.

## Supplementary Information

Supplementary material file1

## Data Availability

The datasets supporting the conclusions of this article and analysis code (R) are available in the OSF repository: https://osf.io/e54py/. Both studies were preregistered.

## Appendix A: Event Description "Living Room"

**Introduction sentences:** The four friends, Arian, Brian, Chris, and Sophie, meet at Brian's apartment. They are in the living room and have ordered Chinese food.

1. The friends sit around a coffee table.
2. The coffee table stands in the middle of the living room.
3. A dark tablecloth is spread out on the couch table.
4. Arian sits on a couch.
5. Arian has black hair.
6. Arian wears a watch.
7. The couch is made of leather.
8. Behind the couch stands a big bookshelf.
9. Next to the bookshelf is a plant.
10. Sophie sits in an armchair.
11. Sophie wears a blue dress.
12. Sophie wears a gray jacket.
13. Next to Sophie sits Chris.
14. Chris takes off his jacket.
15. Chris wears glasses.
16. Chris sits on a wooden chair.
17. Brian walks toward the sofa.
18. Brian wears a red t-shirt and jeans.
19. Brian holds a stack of blue plates in his hands.
20. Brian puts the stack of plates on the table.
21. A plastic box with a transparent lid stands in front of Sophie.
22. Next to the food box stands a coke.
23. Sophie takes a piece of fried vegetables.
24. Sophie throws the vegetable in the air.
25. Chris picks up a food container.
26. Chris eats a piece of the fried vegetables.
27. Chris watches Sophie.
28. Brian stops in front of the couch.
29. Brian sits down.
30. Sophie chews briefly and laughs.
31. Brian informs Chris that he does not like it when Sophie plays with food.
32. Brian takes a plate from the stack.
33. Arian leans forward to the table.
34. Arian holds a take-away food box in his hand.
35. Sophie teases Brian about his attitude.
36. Brian looks at Chris.
37. Chris cleans his glasses.
38. Chris explains to Sophie that Brian is upset.
39. Chris begins to laugh.
40. Sophie turns toward Brian and laughs.

**Outro:** The friends eat their food and enjoy the rest of the evening.

## Appendix B: Confidence integrated into the Content Memory model (Study 1)

We also included confidence as a variable in the model with content memory as preregistered. It showed that confidence in the response predicted the likelihood of a correct answer, *χ*^*2*^(1) = 785.65, *p* < 0.001. Additionally, there was an interaction effect between confidence and discriminability, *χ*^*2*^(2) = 11.73, *p* = 0.003. In the low and high discriminability, confidence was generally lower compared to the control condition. The interaction effect between discriminability and veracity was also still present, *χ*^*2*^(2) = 7.97, *p* = 0.01. However, we did not find a three-way interaction between confidence, veracity, and discriminability, *χ*^*2*^(2) = 0.90, *p* = 0.636 (Table 4).

**Table 4:** Results of study 1 for a generalized mixed-effect model with confidence as interaction effect

Predictor	*Odds ratio*	β	95% CI	Standardized CI	*z*	*p*
*Fixed Effects*						
(Intercept)	5.51	5.51	4.28 – 7.10	4.28 – 7.10	13.21	** < .001**
Discriminability [high]	1.05	1.05	0.75 – 1.48	0.75 – 1.48	0.30	.768
Discriminability [low]	0.92	0.92	0.65 – 1.29	0.65 – 1.29	−0.49	.627
Veracity [false]	1.00	1.00	0.76 – 1.30	0.76 – 1.30	−0.03	.976
Discriminability [high] x Veracity [false]	0.59	0.59	0.41 – 0.84	0.41 − 0.84	−2.91	**.004**
Discriminability [low] x Veracity [false]	0.68	0.68	0.48 – 0.98	0.48 – 0.98	−2.09	**.036**
Confidence	2.08	3.35	1.91 – 2.27	2.90 – 3.87	16.57	** < .001**
Confidence x discriminability [high]	0.88	0.81	0.79 – 0.99	0.67 – 0.99	−2.09	**.037**
Confidence x discriminability [low]	0.86	0.78	0.77 – 0.97	0.65 – 0.94	−2.55	**.011**
Confidence x veracity [false]	1.02	1.04	0.88 – 1.20	0.80 – 1.34	0.29	.771
Confidence x (Discriminability [high] x Veracity [false])	0.90	0.84	0.73 – 1.11	0.59 –1.20	−0.96	.339
Confidence x (Discriminability [low] x Veracity [false])	0.94	0.90	0.77 – 1.15	0.65 – 1.26	−0.60	.550
**Random effects**						
*N*_*participant*_	198					
*N*_*item*_	200					
Observations	7920					

## Study 1: New sentences

Participants could correctly identify each sentence as new or misidentify it as old. We did the same analysis as before but only included the fixed factor discriminability (control vs. low vs. high discriminability), as the new sentences did not have any veracity value. Participant and item IDs were added as a random intercept to the model. The resulting model was submitted to a type III ANOVA (Table 5).

**Table 5:** Results of a generalized mixed-effect model for correct answers for new sentences as dependent variable and discriminability as fixed factor

Predictor	Estimates	β	95% CI	Standardized CI	*t*	*p*
*Fixed Effects*						
(Intercept)	34.89	34.89	22.87 – 53.24	22.87 – 53.24	16.48	< .001
Discriminability [high]	0.56	0.56	0.34 – −0.93	0.34 – 0.93	−2.35	.023
Discriminability [low]	0.53	0.53	0.32 – 0.88	0.32 – 0.88	−2.48	.013
*Random effects*						
*N*_*participant*_	198					
*N*_*item*_	200					
Observations	7,920					

**Veracity memory.** We tested whether the discriminability (control vs. low vs. high discriminability) predicted the likelihood of correctly identifying the new sentences. The model showed a significant difference in memory between the discriminability conditions, *χ*^*2*^(2) = 7.43, *p* = 0.024. Participants in the control condition were better in identifying the new sentences as distractors compared to the low, *χ*^*2*^(1) = 5.75, *p* = 0.016, and high-discriminability condition, *χ*^*2*^(1) = 5.63, *p* = 0.018. There was no difference between the two discriminability conditions, *χ*^*2*^(1) = 0.48, *p* = 0.827.

## Study 2: New sentences

Participants could correctly identify new sentences as new or misidentify them as true or false. We only included the fixed factor discriminability (low vs. high discriminability) in the hierarchical model. Participants and item IDs were again added as a random intercept to the model (Table 6).

**Table 6:** Results of the generalized linear model fitted to Study 2 veracity memory for new sentences with fixed factor discriminability

**Predictor**	**Odds ratio**	**β**	**95% CI**	**Standardized CI**	***z***	***p***
*Fixed effects*						
(Intercept)	2.37	2.37	[1.59 – 3.53]	[1.59 – 3.53]	4.23	< .001
Discriminability [low]	1.17	0.32	[0.69 – 2.00]	[0.69 – 2.00]	0.59	.557
*Random effects*						
*N*_*participant*_	248					
*N*_*item*_	200					
Observations	9,920					

The resulting model was submitted to a type II ANOVA. We additionally fitted a model with the dummy variable classification where the dependent variable answer was transferred into the three possible responses: correct, misidentified as true, and misidentified as false (Table 7).

**Table 7:** Results of the generalized linear model fitted to Study 2 veracity memory for new sentences with fixed factors discriminability and classification

Predictor	Odds ratio	β	95% CI	Standardized CI	*z*	*p*
*Fixed effects*						
(Intercept)	2.25	2.25	[1.99 – 2.55]	[1.99 – 2.55]	12.89	< .001
Discriminability [low]	0.88	0.88	[0.74 – 1.03]	[0.74 – 1.03]	−1.59	.112
Veracity [false]	0.26	0.26	[0.23 – 0.30]	[0.23 – 0.30]	−18.72	< .001
Discriminability [low] x Veracity [false]	0.93	0.93	[0.76 – 1.13]	[0.76 – 1.13]	−0.74	.458
*Random effects*						
*N*_*participant*_	248					
*N*_*item*_	200					
Observations	9,920					

**Veracity memory.** Participants mostly correctly identified new sentences as new, and we did not find a difference between the discriminability conditions, *χ*^*2*^(1) = 0.34, *p* = 0.557.

**Misidentified truth value.** We were also interested in whether new sentences misidentified as old were more likely given the value true or the value false (classification: correct, misidentified as true, misidentified as false). We found a significant interaction effect between classification and discriminability, *χ*^*2*^(1) = 28.76, *p* < 0.001. The probability was significantly higher that new sentences were classified as false than as true, and this effect was stronger for the high-discriminability condition, *z* = −37.91, *p* < 0.001, than for the low-discriminability condition, *z* = −4.18, *p* < 0.001.

Thus, when the new sentences were given a truth value, it was more likely that they received the value *false* than *true*, and this effect was stronger in the high-discriminability condition.

## Footnotes

1 Participants who did not pass the color test were still paid for their participation.

2 In the preregistration, the confidence hypothesis was misstating *confidence*, when it was supposed to state *veracity*. Thus, we were interested in the interaction of veracity and discriminability (as in line with Study 1), not confidence and discriminability.

3 Results from this analysis can be found in the Supplement Material.

## References

[CR1] Allport, G. W., & Postman, L. (1946). An analysis of rumor. *Public Opinion Quarterly,**10*(4), 501–517.

[CR2] Alter, A. L. (2013). The Benefits of Cognitive Disfluency. *Current Directions in Psychological Science,**22*(6), 437–442. 10.1177/0963721413498894

[CR3] Alter, A. L., & Oppenheimer, D. M. (2009). Uniting the Tribes of Fluency to Form a Metacognitive Nation. *Personality and Social Psychology Review,**13*(3), 219–235. 10.1177/1088868309341564 19638628 10.1177/1088868309341564

[CR4] Braasch, J. L., & Kessler, E. D. (2021). Working toward a theoretical model for source comprehension in everyday discourse. *Discourse Processes,**58*(5–6), 449–467.

[CR5] Brashier, N. M., Pennycook, G., Berinsky, A. J., & Rand, D. G. (2021). Timing matters when correcting fake news. *Proceedings of the National Academy of Sciences,**118*(5), Article e2020043118. 10.1073/pnas.2020043118 10.1073/pnas.2020043118 PMC7865139 33495336

[CR6] Clayton, K., Blair, S., Busam, J. A., Forstner, S., Glance, J., Green, G., Kawata, A., Kovvuri, A., Martin, J., Morgan, E., et al. (2020). Real solutions for fake news? Measuring the effectiveness of general warnings and fact-check tags in reducing belief in false stories on social media. *Political Behavior,**42*(4), 1073–1095.

[CR7] Dechêne, A., Stahl, C., Hansen, J., & Wänke, M. (2010). The truth about the truth: A meta-analytic review of the truth effect. *Personality and Social Psychology Review,**14*(2), 238–257. 10.1177/1088868309352251 20023210 10.1177/1088868309352251

[CR8] Ecker, U. K. H., Lewandowsky, S., Cook, J., Schmid, P., Fazio, L. K., Brashier, N., Kendeou, P., Vraga, E. K., & Amazeen, M. A. (2022). The psychological drivers of misinformation belief and its resistance to correction. *Nature Reviews Psychology,**1*(1), 13–29. 10.1038/s44159-021-00006-y

[CR9] Eitel, A., Kühl, T., Scheiter, K., & Gerjets, P. (2014). Disfluency Meets Cognitive Load in Multimedia Learning: Does Harder-to-Read Mean Better-to-Understand? *Applied Cognitive Psychology,**28*(4), 488–501. 10.1002/acp.3004

[CR10] Fernandez, M., & Alani, H. (2018). Online misinformation: Challenges and future directions. *Companion of the The Web Conference 2018 on The Web Conference 2018 - WWW '18*, 595–602. 10.1145/3184558.3188730

[CR11] Fleming, S. M. (2024). Metacognition and Confidence: A Review and Synthesis. *Annual Review of Psychology*, *75*(1), annurev-psych-022423–032425. 10.1146/annurev-psych-022423-032425 10.1146/annurev-psych-022423-032425 37722748

[CR12] Flesch, R. (1979). *How to write plain English*. https://pages.stern.nyu.edu/~wstarbuc/Writing/Flesch.htm

[CR13] George, J., Gerhart, N., & Torres, R. (2021). Uncovering the truth about fake news: A research model grounded in multi-disciplinary literature. *Journal of Management Information Systems,**38*(4), 1067–1094. 10.1080/07421222.2021.1990608

[CR14] Gilbert, D. T., Tafarodi, R. W., & Malone, P. S. (1993). You can’t not believe everything you read. *Journal of Personality and Social Psychology,**65*(2), 221–233. 10.1037/0022-3514.65.2.221 8366418 10.1037//0022-3514.65.2.221

[CR15] Grady, R. H., Ditto, P. H., & Loftus, E. F. (2021). Nevertheless, partisanship persisted: Fake news warnings help briefly, but bias returns with time. *Cognitive Research: Principles and Implications,**6*(1), 52. 10.1186/s41235-021-00315-z 34297248 10.1186/s41235-021-00315-z PMC8299168

[CR16] Hassan, A., & Barber, S. J. (2021). The effects of repetition frequency on the illusory truth effect. *Cognitive Research: Principles and Implications,**6*(1), 38. 10.1186/s41235-021-00301-5 33983553 10.1186/s41235-021-00301-5 PMC8116821

[CR17] Kimball, D. R., & Bjork, R. A. (2002). Influences of intentional and unintentional forgetting on false memories. *Journal of Experimental Psychology: General,**131*(1), 116–130. 10.1037/0096-3445.131.1.116 11900099 10.1037//0096-3445.131.1.116

[CR18] Kozyreva, A., Lorenz-Spreen, P., Herzog, S. M., Ecker, U. K. H., Lewandowsky, S., Hertwig, R., Ali, A., Bak-Coleman, J., Barzilai, S., Basol, M., Berinsky, A. J., Betsch, C., Cook, J., Fazio, L. K., Geers, M., Guess, A. M., Huang, H., Larreguy, H., Maertens, R., … Wineburg, S. (2024). Toolbox of individual-level interventions against online misinformation. *Nature Human Behaviour*, *8*(6), 1044–1052. 10.1038/s41562-024-01881-0 10.1038/s41562-024-01881-0 38740990

[CR19] Leahy, W., & Sweller, J. (2008). The imagination effect increases with an increased intrinsic cognitive load. *Applied Cognitive Psychology,**22*(2), 273–283. 10.1002/acp.1373

[CR20] Lewandowsky, S., Ecker, U. K., Seifert, C. M., Schwarz, N., & Cook, J. (2012). Misinformation and its correction: Continued influence and successful debiasing. *Psychological Science in the Public Interest,**13*(3), 106–131. 26173286 10.1177/1529100612451018

[CR21] Loftus, E. F. (2005). Planting misinformation in the human mind: A 30-year investigation of the malleability of memory: Figure 1. *Learning & Memory,**12*(4), 361–366. 10.1101/lm.94705 16027179 10.1101/lm.94705

[CR22] Marsh, E. J., & Fazio, L. K. (2006). Learning errors from fiction: Difficulties in reducing reliance on fictional stories. *Memory & Cognition,**34*(5), 1140–1149. 10.3758/BF03193260 17128612 10.3758/bf03193260

[CR23] Mayo, R. (2024). Trust or distrust? Neither! The right mindset for confronting disinformation. *Current Opinion in Psychology,**56*, Article 101779. 10.1016/j.copsyc.2023.101779 38134524 10.1016/j.copsyc.2023.101779

[CR24] Nadarevic, L., & Erdfelder, E. (2013). Spinoza’s error: Memory for truth and falsity. *Memory & Cognition,**41*(2), 176–186. 10.3758/s13421-012-0251-z 22972664 10.3758/s13421-012-0251-z

[CR25] Nadarevic, L., & Erdfelder, E. (2019). More evidence against the Spinozan model: Cognitive load diminishes memory for “true” feedback. *Memory & Cognition,**47*(7), 1386–1400. 10.3758/s13421-019-00940-6 31215012 10.3758/s13421-019-00940-6

[CR26] Nadarevic, L., Symeonidou, N., & Kias, A. (2022). In Colore Veritas? Color effects on the speed and accuracy of true/false responses. *Psychological Research Psychologische Forschung,**86*(3), 919–936. 10.1007/s00426-021-01528-z 34050785 10.1007/s00426-021-01528-z PMC8942928

[CR27] Oppenheimer, D. M. (2008). The secret life of fluency. *Trends in Cognitive Sciences,**12*(6), 237–241. 10.1016/j.tics.2008.02.014 18468944 10.1016/j.tics.2008.02.014

[CR28] Pantazi, M., Kissine, M., & Klein, O. (2018). The power of the truth bias: False information affects memory and judgment even in the absence of distraction. *Social Cognition,**36*(2), 167–198.

[CR29] Pennycook, G., Bear, A., Collins, E. T., & Rand, D. G. (2020). The Implied Truth Effect: Attaching Warnings to a Subset of Fake News Headlines Increases Perceived Accuracy of Headlines Without Warnings. *Management Science,**66*(11), 4944–4957. 10.1287/mnsc.2019.3478

[CR30] Reber, R., & Schwarz, N. (1999). Effects of perceptual fluency on judgments of truth. *Consciousness and Cognition,**8*(3), 338–342. 10.1006/ccog.1999.0386 10487787 10.1006/ccog.1999.0386

[CR31] Richter, T., Schroeder, S., & Wöhrmann, B. (2009). You don’t have to believe everything you read: Background knowledge permits fast and efficient validation of information. *Journal of Personality and Social Psychology,**96*(3), 538–558. 10.1037/a0014038 19254102 10.1037/a0014038

[CR32] Rummer, R., Schweppe, J., & Schwede, A. (2016). Fortune is fickle: Null-effects of disfluency on learning outcomes. *Metacognition and Learning,**11*(1), 57–70. 10.1007/s11409-015-9151-5

[CR33] Shin, J., Jian, L., Driscoll, K., & Bar, F. (2018). The diffusion of misinformation on social media: Temporal pattern, message, and source. *Computers in Human Behavior,**83*, 278–287. 10.1016/j.chb.2018.02.008

[CR34] Street, C. N. H., & Kingstone, A. (2017). Aligning Spinoza with Descartes: An informed Cartesian account of the truth bias. *British Journal of Psychology,**108*(3), 453–466. 10.1111/bjop.12210 27511287 10.1111/bjop.12210

[CR35] Unkelbach, C. (2007). Reversing the truth effect: Learning the interpretation of processing fluency in judgments of truth. *Journal of Experimental Psychology: Learning, Memory, and Cognition,**33*(1), 219–230. 10.1037/0278-7393.33.1.219 17201563 10.1037/0278-7393.33.1.219

[CR36] Wixted, J. T., Mickes, L., & Fisher, R. P. (2018). Rethinking the reliability of eyewitness memory. *Perspectives on Psychological Science,**13*(3), 324–335. 10.1177/1745691617734878 29716454 10.1177/1745691617734878

[CR37] Zwaan, R. A., & Radvansky, G. A. (1998). Situation models in language comprehension and memory. *Psychological Bulletin,**123*(2), 162–185. 10.1037/0033-2909.123.2.162 9522683 10.1037/0033-2909.123.2.162

