# Supplementary material for: Processing of veracity cues: how processing difficulty affects the memory of event description and judgment of confidence
Source: Cogn Res Princ Implic. 2025 May 28;10:22. doi: 10.1186/s41235-025-00629-2 (PMC12119432; doi:10.1186/s41235-025-00629-2)
Supplement: Supplementary file 1 — Supplementary material file1 [file 41235_2025_629_MOESM1_ESM.docx]

**Supplement Material**

**Veracity Memory Model with coded variable classification**

We additionally preregistered the analysis in which the correct answer was separated by classification following Pantazi and colleagues.

Participants could correctly identify each sentence as true-labeled or false-labeled or misidentify it as new. Additionally, true-labeled sentences could be confused as false and false-labeled sentences as true. Thus, responses can be sorted into three classifications: correct, confused, and misidentified. We treated this classification factor as repeated measurement equivalent to the analysis procedure of Pantazi and colleagues. Thus, every item was in the data set three times for all participants, and the response was transferred to 1 based on their answer in that classification factor. For example, if a sentence had the veracity cue *false* and the participant responded *true*, the response was categorized as 1 on the classification level confused. In contrast, on the classification levels *correct* or *misidentified*, it was categorized as 0.

To account for individual variations, we included participants' and item IDs as random intercepts in the models. Both resultant models (for veracity memory and confidence) were submitted to a Type II ANOVA for analysis.

**Veracity memory.** As a first analysis, we tested how veracity memory is affected by discriminability, veracity cue, and classification. However, we did not find a three-way interaction between classification, discriminability, and veracity cue, *χ^2^*(2) = 2.17, *p* = .338. Thus, the classification pattern for true-labeled and false-labeled sentences did not differ between the low and high discriminability conditions. However, the model showed a significant interaction effect between veracity cue and classification, *χ^2^*(2) = 1612.97, *p* < .001. False-labeled sentences were significantly less often correctly identified than true-labeled sentences, OR = 0.29, 95% CI = [-0.25, 0.33], *p* < .001. Further, false-labeled sentences were more often confused, OR = 15.25, 95% CI = [12.57, 18.51], *p* < .001, and more often misidentified compared to true-labeled sentences, OR = 3.36, 95% CI = [2.69, 4.19], *p* < .001. These effects were independent of the discriminability condition (see Table S1).

Additionally, the model showed a significant interaction effect between classification and discriminability, *χ^2^*(2) = 20.26, *p* < .001. Participants in the low discriminability condition more often misidentified sentences, OR = 1.33, 95% CI = [1.13, 1.55], *p* < .001, and less often correctly identified sentences compared to the high discriminability condition, OR = 0.89, 95% CI = [0.81, 0.98], *p =* .015. Both conditions equally often confused sentences' veracity OR = 1.16, 95% CI = [0.99, 1.13], *p* = .061. Thus, the increased difficulty to discriminate true-labeled and false-labeled sentences affected the general memory performance but did not result in a higher confusion rate of the veracity cue.

**Table S1.**

*Results of the Generalized Linear Model fitted to Study 2: Veracity Memory.*

| **Predictor** | **Odds ratio** | **β** | **95% CI** | **Standardized CI** | ***z*** | ***p*** |
| --- | --- | --- | --- | --- | --- | --- |
| **Fixed effects** |  |  |  |  |  |  |
| (Intercept) | 2.12 | 2.12 | 1.98 – 2.27 | 1.98 – 2.27 | 21.43 | **<0.001** |
| Classification [confused] | 0.09 | 0.09 | 0.08 – 0.10 | 0.08 – 0.10 | -42.20 | **<0.001** |
| Classification [misidentified] | 0.09 | 0.09 | 0.08 – 0.10 | 0.08 – 0.10 | -42.76 | **<0.001** |
| Veracity cue [false] | 0.29 | 0.29 | 0.25 – 0.33 | 0.25 – 0.33 | -18.21 | **<0.001** |
| Discriminability [low] | 0.89 | 0.89 | 0.81 – 0.98 | 0.81 – 0.98 | -2.43 | **0.015** |
| Classification [confused] × Veracity cue [false] | 15.25 | 15.25 | 12.57 – 18.51 | 12.57 – 18.51 | 27.58 | **<0.001** |
| Classification [misidentified] × Veracity cue [false] | 3.36 | 3.36 | 2.69 – 4.19 | 2.69 – 4.19 | 10.72 | **<0.001** |
| Classification [confused] × discriminability [low] | 1.16 | 1.16 | 0.99 – 1.36 | 0.99 – 1.36 | 1.88 | 0.061 |
| Classification [misidentified] × Discriminability [low] | 1.33 | 1.33 | 1.13 – 1.55 | 1.13 – 1.55 | 3.56 | **<0.001** |
| Veracity cue [false] × Discriminability [low] | 0.92 | 0.92 | 0.76 – 1.11 | 0.76 – 1.11 | -0.86 | 0.390 |
| (Classification [confused] × Veracity cue [false]) × discriminability [low] | 1.21 | 1.21 | 0.92 – 1.60 | 0.92 – 1.60 | 1.37 | 0.171 |
| (Classification [misidentified] × Veracity cue [false]) × Discriminability [low] | 1.01 | 1.01 | 0.74 – 1.38 | 0.74 – 1.38 | 0.08 | 0.936 |
| **Random effects** | | | | | | |
| *N_participant_* | 248 | | | | | |
| *N_item_* | 200 | | | | | |
| Observations | 29,760 | | | | | |
